# Comparative Efficiency of Gene-Activated Matrices Based on Chitosan Hydrogel and PRP Impregnated with *BMP2* Polyplexes for Bone Regeneration

**DOI:** 10.3390/ijms232314720

**Published:** 2022-11-25

**Authors:** Irina Alekseevna Nedorubova, Tatiana Borisovna Bukharova, Viktoria Olegovna Mokrousova, Maria Aleksandrovna Khvorostina, Andrey Vyacheslavovich Vasilyev, Andrey Anatolevich Nedorubov, Timofei Evgenevich Grigoriev, Yuriy Dmitrievich Zagoskin, Sergei Nicolaevich Chvalun, Sergey Ivanovich Kutsev, Dmitry Vadimovich Goldshtein

**Affiliations:** 1 Research Centre for Medical Genetics, 115478 Moscow, Russia; 2 Central Research Institute of Dental and Maxillofacial Surgery, 119021 Moscow, Russia; 3 Institute of Photon Technologies of Federal Scientific Research Centre “Crystallography and Photonics”, Russian Academy of Sciences, 108840 Moscow, Russia; 4 Institute of Translational Medicine and Biotechnology, Sechenov University, 119991 Moscow, Russia; 5 NRC “Kurchatov Institute”, 123182 Moscow, Russia

**Keywords:** gene-activated matrices, plasmid DNA, polylactide granules, chitosan, platelet rich plasma, bone regeneration

## Abstract

Gene therapy is one of the most promising approaches in regenerative medicine. Gene-activated matrices provide stable gene expression and the production of osteogenic proteins in situ to stimulate osteogenesis and bone repair. In this study, we developed new gene-activated matrices based on polylactide granules (PLA) impregnated with *BMP2* polyplexes and included in chitosan hydrogel or PRP-based fibrin hydrogel. The matrices showed high biocompatibility both in vitro with mesenchymal stem cells and in vivo when implanted intramuscularly in rats. The use of porous PLA granules allowed the inclusion of a high concentration of polyplexes, and the introduction of the granules into hydrogel provided the gradual release of the plasmid constructs. All gene-activated matrices showed transfecting ability and ensured long-term gene expression and the production of target proteins in vitro. At the same time, the achieved concentration of BMP-2 was sufficient to induce osteogenic differentiation of MSCs. When implanted into critical-size calvarial defects in rats, all matrices with *BMP2* polyplexes led to new bone formation. The most significant effect on osteoinduction was observed for the PLA/PRP matrices. Thus, the developed gene-activated matrices were shown to be safe and effective osteoplastic materials. PLA granules and PRP-based fibrin hydrogel containing *BMP2* polyplexes were shown to be the most promising for future applications in bone regeneration.

## 1. Introduction

Gene therapy is one of the most promising approaches for bone regeneration. This method ensures local, controlled and sustained production of therapeutic proteins, simulating the natural wound-healing process [1,2]. Bone morphogenetic proteins (BMPs) are active participants in natural osteogenesis [3,4], and BMP-2, in turn, is the most effective osteoinductor that promotes the differentiation of mesenchymal stem cells (MSCs) into osteoblasts [5]. Viral and nonviral methods for target gene delivery are used in gene therapy. Viral vectors provide high transduction efficiency because of the natural mechanism that enables entry into cells. To date, over 2000 clinical trials of viral gene therapy have been completed worldwide [6]. However, the use of viral vectors is limited because of the risk of insertional mutagenesis [7] and immunogenicity [8]. Consequently, more attention is paid to nonviral vectors as they are safer [9].

Genetic constructs are impregnated into biocompatible and bioresorbable materials to obtain so-called gene-activated matrices to ensure targeted delivery to the bone defect area and prolonged release [10]. Materials must have sufficient elasticity and strength to maintain shape, and they must be able to integrate with the surrounding native bone during regeneration [11]. Many synthetic and natural polymers have these properties and are therefore attractive for biomedical applications.

Synthetic polymers such as polylactic acid (PLA), polyglycolic acid (PGA) and poly (lactic-co-glycolic) acid (PLGA), which have good biocompatibility, mechanical strength, functional group availability and surface modification, are widely used in bone grafting for various medical purposes [12]. Among them, PLA is considered the most promising FDA-approved material for use in clinical medicine [13]. PLA undergoes hydrolytic cleavage to form lactic acid, which is naturally metabolized in human organisms [14]. PLA scaffolds have been used in ex vivo gene therapy for the intramuscular implantation of MSCs transduced with an adeno-associated virus carrying the *BMP2* gene [15]. Highly porous granules can be obtained from PLA, providing vascular proliferation, migration of osteogenic progenitor cells and efficient transport of metabolites and substances [16]. In addition, the large surface area inside PLA granules allows them to be impregnated with plasmid constructs at high concentrations. The disadvantage of PLA is poor wettability and brittleness. Mixing PLA granules with hydrogels makes it possible to increase the hydrophilicity of the polymer and obtain a strong elastic material. Such combinations display optimal biological and physical and mechanical properties and are of great interest for regenerative medicine.

Chitosan or platelet-rich plasma (PRP)-based fibrin show promise for use as hydrogels. It has been shown that chitosan hydrogel has antibacterial properties and promotes wound healing [17]. The properties of chitosan hydrogel can be varied by physical or chemical exposure [18]. In addition, chitosan, as a polycationic molecule, can be a transfecting agent for gene delivery into cells [19,20], which makes it a suitable candidate for use in the creation of gene-activated matrices. Fibrin hydrogels based on the patient’s PRP allow the acquisition of autogenous materials with high biocompatibility for tissue regeneration. Moreover, numerous growth factors contained in PRP are released when the platelets are activated by thrombin and calcium, stimulating angiogenesis and mitotic and metabolic activity of cells involved in regeneration [21].

The aim of this study was to develop gene-activated matrices based on PLA granules, chitosan hydrogel and PRP-based fibrin hydrogel impregnated with *BMP2* polyplexes, which have a high osteoinductive effect in vitro and in vivo.

## 2. Results

### 2.1. Biocompatibility of Matrices

**Figure 1:**
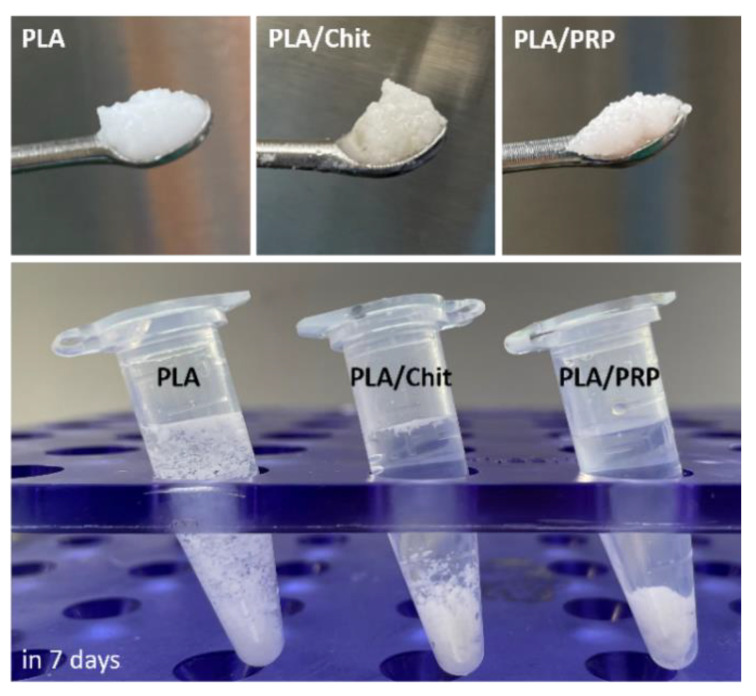
Matrices based on PLA granules, PLA/Chit hydrogel and PLA/PRP hydrogel in saline solution.

Porous PLA granules were used as the main component of the carrier material. PLA granules were mixed with chitosan hydrogel (Chit) or PRP-based fibrin hydrogel (PRP) to obtain shaped matrices. As a result, new matrices based on PLA, PLA/Chit and PLA/PRP were obtained (Figure 1). The PLA/PRP matrices were the densest materials capable of maintaining their shape in saline for a long period.

**Figure 2:**
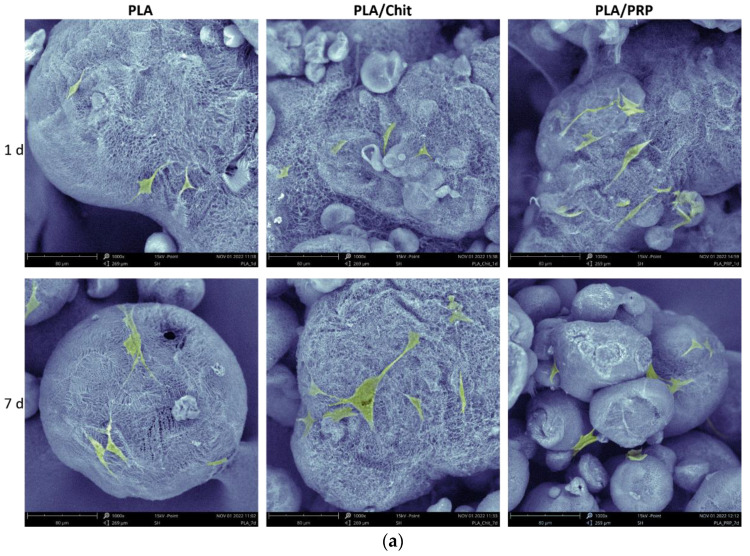

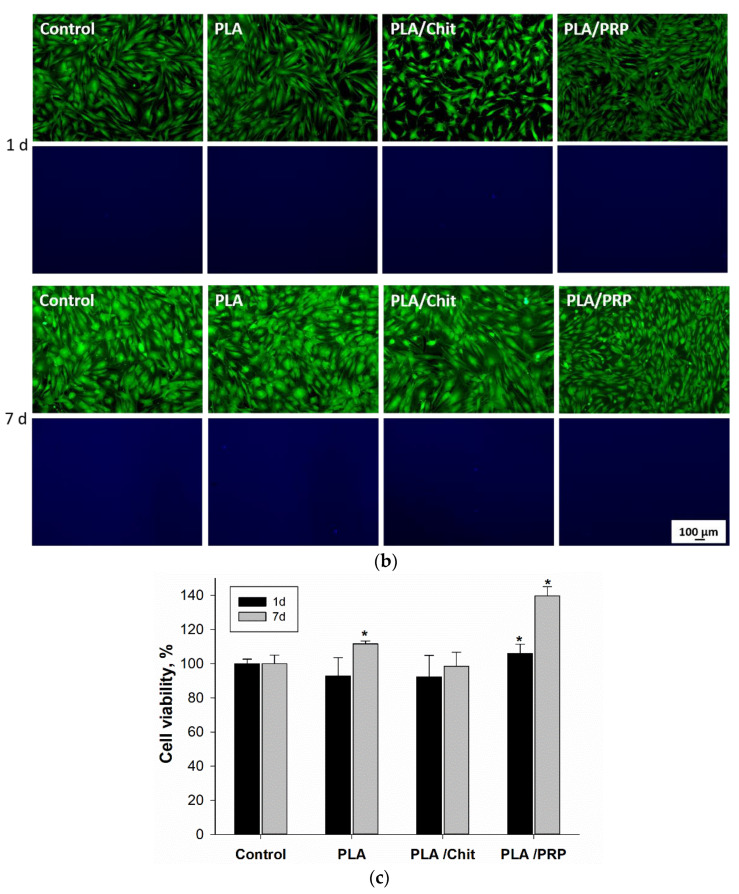
In vitro cytocompatibility of matrices: (**a**) adhesion of MSCs to PLA granules, SEM; (**b**) assessment of cytotoxicity of matrices using fluorescent microscopy of live MSCs stained with calcein AM (green) and dead cells stained with DAPI (blue); and (**c**) viability assessment of MSCS at 1 and 7 days after incubation with the matrices, MTT-test. * *p* < 0.05 (relative to control).

Detailed analysis of the adhesion of MSCs to matrices after 24 h using SEM showed that cells were attached at high density to the porous surface of the PLA granules (Figure 2a). The MSCs retained their characteristic morphology—spread polygonal and fibroblast-like cells were observed. The cells adhered tightly to the material surface.

MTT test and staining for live and dead cells showed that all the studied matrices had high cytocompatibility (Figure 2b,c). After 1 and 7 days, most of the cells were alive and stained with calcein AM, and hardly any dead cells stained with DAPI were observed (Figure 2b). The viability of the MSCs 7 days after incubation with PLA, PLA/Chit and PLA/PRP was 111.7 ± 1.6%, 98.5 ± 8.2% and 139.6 ± 5.4%, respectively, compared with the control (Figure 2c).

**Figure 3:**
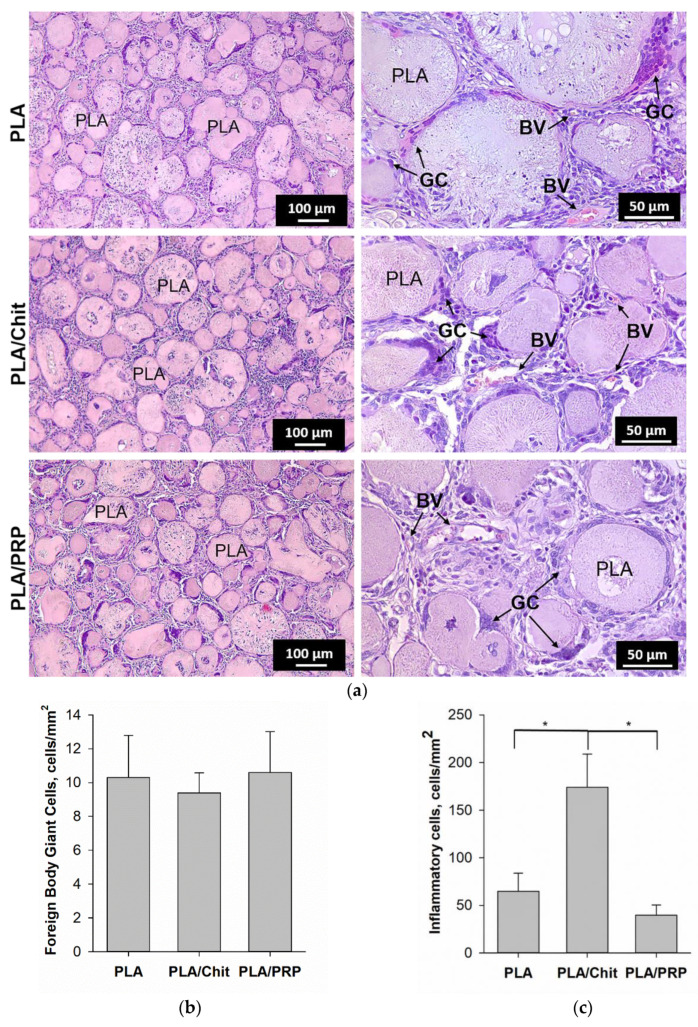
In vivo biocompatibility of matrices 28 days after intramuscular implantation: (**a**) tissue sections of the implantation area, H&E staining. Light microscopy. BV—blood vessel, GC—giant cells; (**b**) quantitative assessment of the resorption degree of the matrices; and (**c**) quantitative assessment of the degree of inflammation. * *p* < 0.05 (comparison between the groups).

The formation of granulation tissue with young fibroblasts, full-blooded vessels and small amounts of leukocytes, lymphocytes and giant cells of foreign bodies surrounding the PLA granules was observed 28 days after intramuscular implantation of the matrices (Figure 3a). Collagen fibers of connective tissue replaced chitosan hydrogel and PRP-based fibrin hydrogel. PLA granules were resorbed by giant cells of foreign bodies. There was no statistically significant difference in the number of giant cells of foreign bodies for the different matrices and, accordingly, the intensity of resorption of PLA granules (Figure 3b). For PLA/Chit, the total number of inflammatory cells was 174 ± 35 per mm^2^, which was statistically significantly higher than the number for PLA (65 ± 19 per mm^2^) and PLA/PRP (40 ± 11 per mm^2^) (Figure 3c). However, none of the matrices studied showed signs of severe acute exudative inflammation.

### 2.2. Transfecting Ability of the Matrices

**Figure 4:**
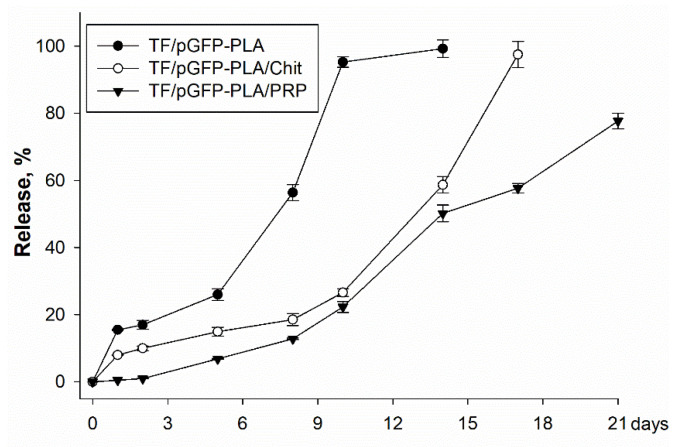
Release of TF/pGFP polyplexes from matrices, spectrophotometry.

TF/pGFP polyplexes were released from the matrices gradually, but the rates of release were significantly different (Figure 4). For PLA, 16% of polyplexes were released on the first day, and 95% were released on the 10th day. Release from PLA/Chit was slower, with 98% of polyplexes observed on the 17th day. Release from PLA/PRP only started on the 5th day (7%), and after 21 days, 78% of the polyplexes were detected.

**Figure 5:**
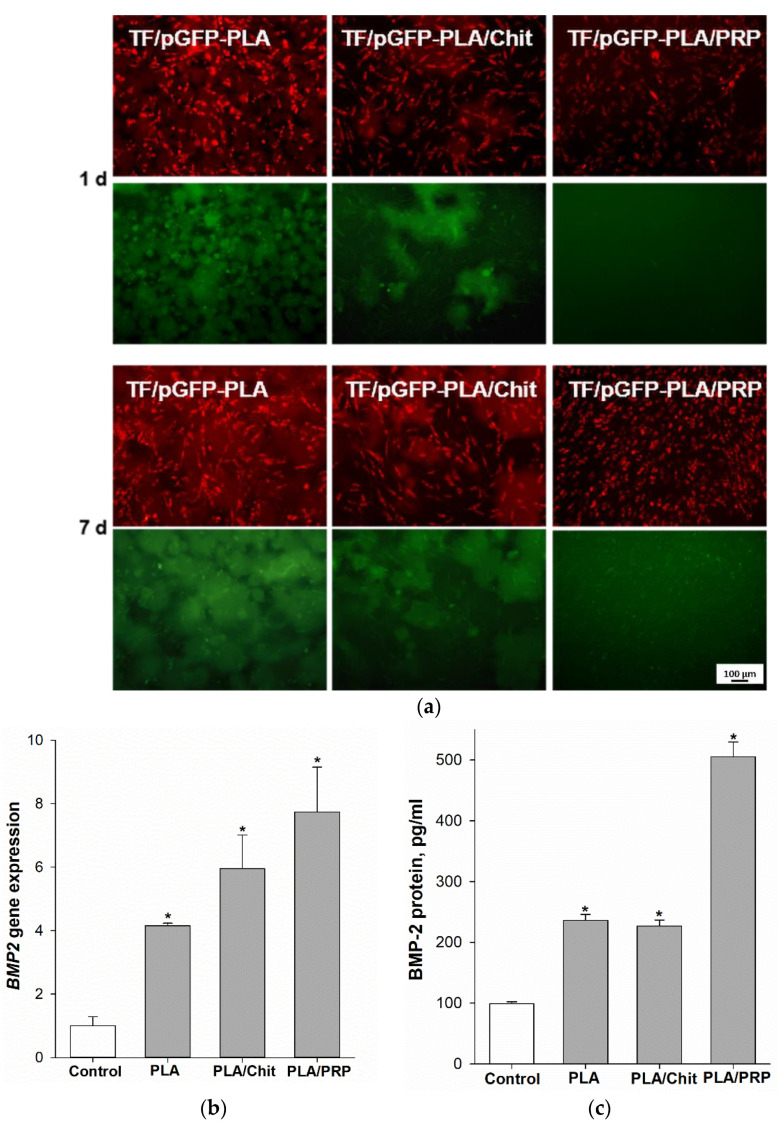
Transfecting ability of gene-activated matrices: (**a**) the ability of TF/pGFP-Matrices to transfect MSCs (green) prestained with PKH-26 (red), fluorescent microscopy; (**b**) relative *BMP2* gene expression 14 days after incubation of MSCs with TF/pBMP2-Matrices, RT-PCR; and (**c**) BMP-2 protein production 14 days after incubation of MSCs with TF/pBMP2-Matrices, ELISA. * *p* < 0.05 (relative to control without matrices).

The presence of MSCs producing GFP protein confirmed the transfecting ability of the obtained matrices impregnated with the TF/pGFP polyplexes (Figure 5a). GFP production in cells was observed after just 1 day of incubation for the PLA and PLA/Chit matrices, and the number of transfected cells increased by day 7. Because of the slower release of polyplexes from PLA/PRP matrices, cell transfection began by day 7.

Comparative analysis of the transfecting efficiency of MSCs using different matrices impregnated with TF/pBMP2 polyplexes was carried out by assessing the levels of *BMP2* gene expression and BMP-2 protein production. Fourteen days after incubation, compared with the negative control, increases were observed in *BMP2* gene expression (4.2 ± 0.1 times for TF/pBMP2-PLA, 5.9 ± 1.1 times for TF/pBMP2-PLA/Chit and 7.7 ± 1.4 times for TF/pBMP2-PLA/PRP) (Figure 5b), and in BMP-2 protein production (for TF/pBMP2-PLA 236.2 ± 9.8 pg/mL, for TF/pBMP2-PLA/Chit 226.9 ± 9.9 pg/mL and 505.4 ± 24.1 pg/mL for TF/pBMP2-PLA/PRP) (Figure 5c).

### 2.3. Osteoinductive Properties of Matrices In Vitro

**Figure 6:**
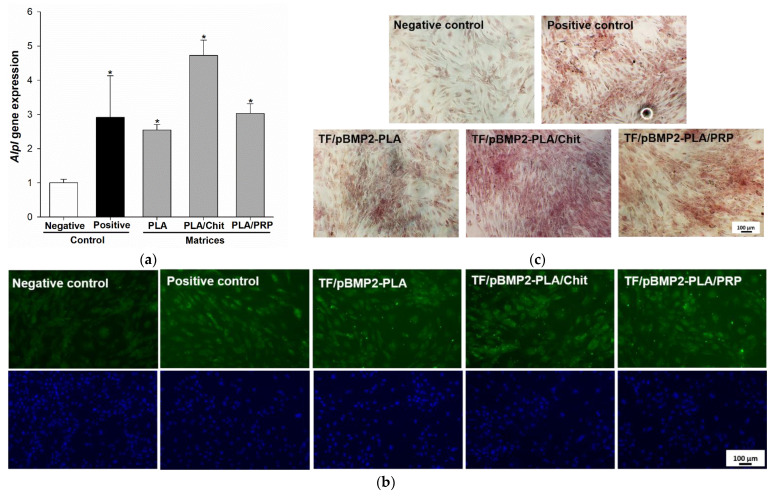
Osteoinductive properties of TF/pBMP2-Matrices 14 days after incubation with MSCs: (**a**) relative *Alpl* gene expression, RT-PCR. * *p* < 0.05 (relative to negative control); (**b**) Alpl protein production (green), nuclei (blue) ICH; and (**c**) ECM mineralization of MSCs, alizarin red staining.

It was shown that the incubation of MSCs for 14 days with gene-activated matrices impregnated with TF/pBMP2 polyplexes led to a significant increase in alkaline phosphatase gene (*Alpl*) expression (2.5 ± 0.2 times for TF/pBMP2-PLA, 4.7 ± 0.5 times for TF/pBMP2-PLA/Chit and 3.0 ± 0.3 times for TF/pBMP2-PLA/PRP) compared to the negative control (Figure 6a) and Alpl protein production (Figure 6b). In addition, the TF/pBMP2-Matrices induced an increase in the mineralization of the extracellular matrix (ECM) of the MSCs (Figure 6c).

### 2.4. Osteoinductive Properties of Matrices In Vivo

**Figure 7:**
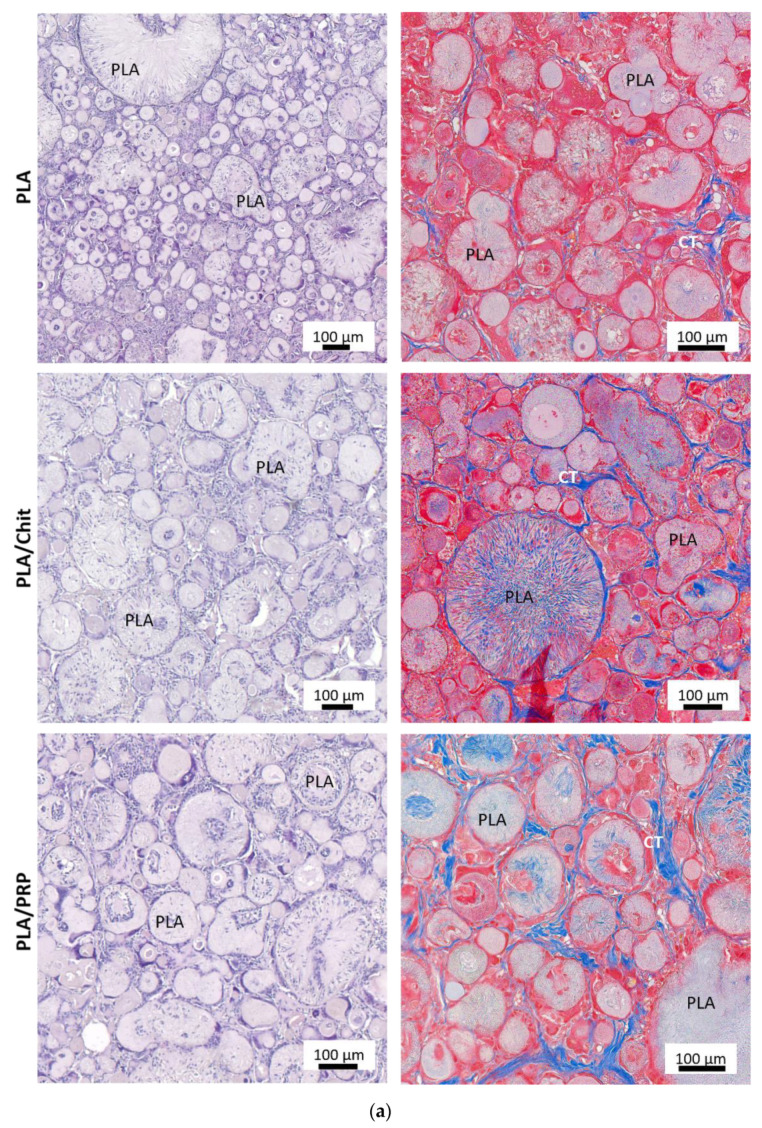

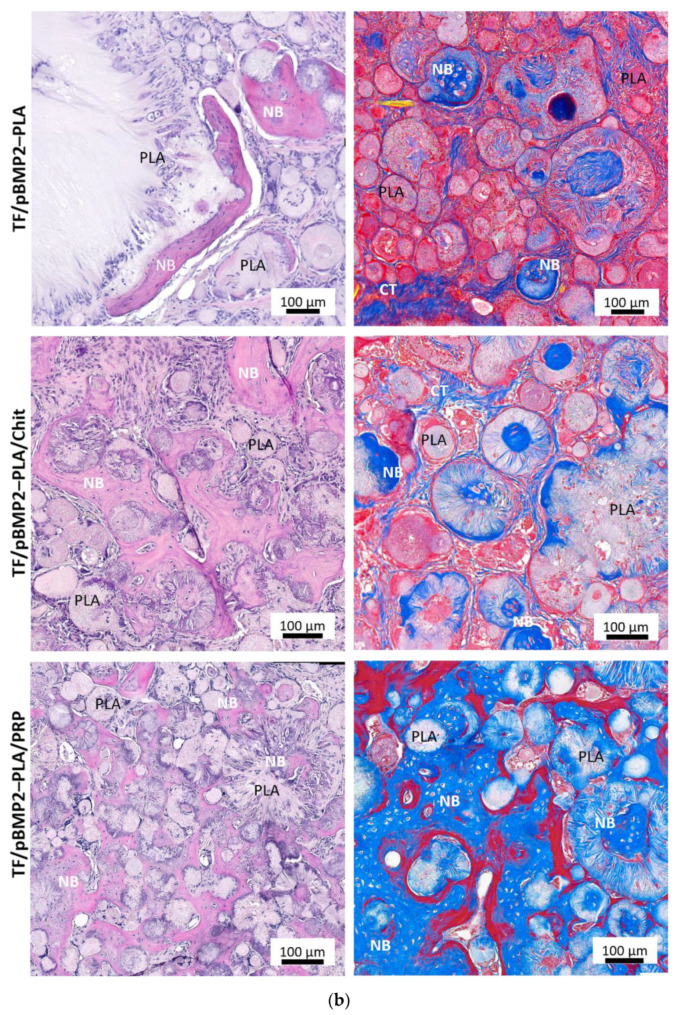
Critical-size calvarial bone defect regeneration 56 days after implantation of the matrices: (**a**) nonactivated matrices; (**b**) TF/BMP2-Matrices. H&E and Masson’s trichrome staining. Light microscopy. CT—connective tissue, NB—newly formed bone.

In all samples, there were no signs of inflammation and partial replacement of biodegradable matrices was observed: chitosan hydrogel and PRP-based fibrin hydrogel were completely replaced by granulation tissue, and PLA granules were resorbed by giant cells of foreign bodies. However, the intensity of resorption for the nonactivated matrices was lower compared with the gene-activated matrices (Figure 7).

**Figure 8:**
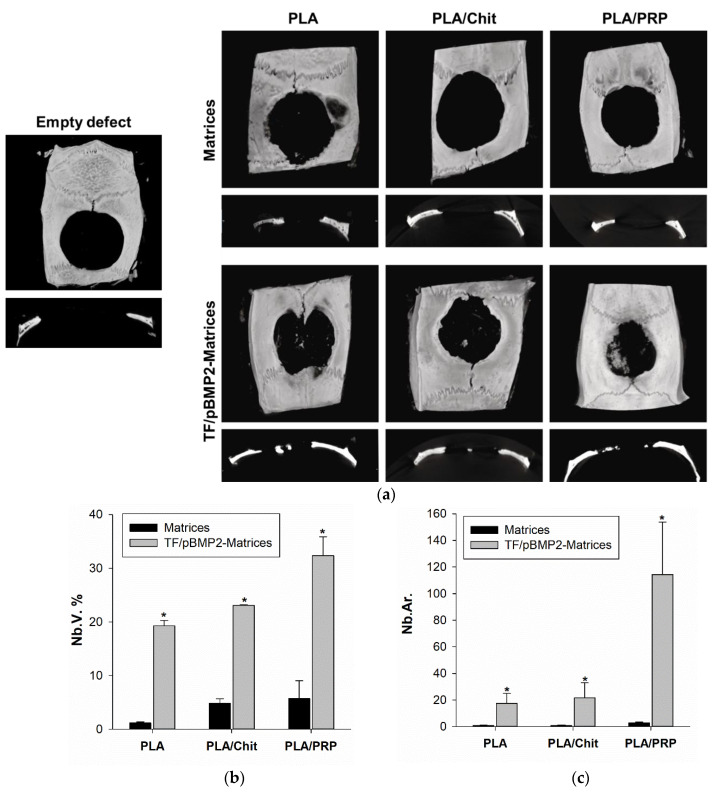
Critical-size bone defect regeneration in rats: (**a**) micro-CT data of critical-size calvarial defect; (**b**) the volume of newly formed bone (Nb.V.) measured using micro-CT; and (**с**) the area of newly formed bone (Nb.Ar.) inside the defect measured using histomorphometrical analysis. * *p* < 0.05 (relative to nonactivated matrices).

In the control groups without genetic constructs, the volume fraction of newly formed bone tissue (NB.V.%) was 1.2 ± 0.2% for PLA, 4.9 ± 0.8% for PLA/Chit and 5.7 ± 3.3% for PLA/PRP (Figure 8b). For PLA and PLA/Chit, there were no statistically significant differences relative to an empty defect (2.6 ± 1.5%).

Upon implantation of gene-activated matrices around and inside the PLA granules, young bone tissue containing many young osteocytes was found. Micro-CT results confirmed a significant difference in the stimulation of osteohistogenesis by gene-activated matrices (Figure 8a) and histomorphometric analysis detected newly formed bone tissue inside the defect (Figure 8b,c). New bone formation was observed mainly from the side of the maternal bone after 8 weeks for all groups. The NB.V.% was 19.3 ± 1.0% for TF/pBMP2-PLA, 23.1 ± 0.1% for TF/pBMP2-PLA/Chit and 32.4 ± 3.5% for TF/pBMP2-PLA/PRP (Figure 8b).

The area of newly formed bone tissue inside the defect (NB.Ar.), estimated histomorphometrically for gene-activated matrices based on TF/pBMP2-PLA/PRP, was 114 ± 40 times higher than for nonactivated PLA/PRP. For TF/pBMP2-PLA/Chit and TF/pBMP2-PLA, it was 21 ± 11 and 17 ± 8 times greater, respectively, than for the corresponding nonactivated matrices (Figure 8c).

## 3. Discussion

When developing gene therapy based on plasmid vectors for bone tissue regeneration, it is important to ensure highly efficient cell transfection to achieve a therapeutic concentration of the osteoinductor in the area of the defect. Transfecting agents that contribute to the successful delivery of plasmids into cells are able to compact plasmids and reduce their negative charge for easy transfer through the cell membrane [1,2]. In our previous work, we showed that when comparing two polycationic molecules, Turbofect (TF) transfected MSCs more efficiently than polyethyleneimine (PEI). In addition, the TaqRFP-N-BMP2 plasmid created for our study could provide a higher level of expression of the target *BMP2* gene in MSCs compared with frequently used constructs based on the pcDNA3 vector. As a result, the composition of polyplexes for efficient cell transfection based on the TaqRFP-N-BMP2 vector and the transfecting agent TF was determined [22].

To ensure targeted delivery of the plasmid DNA and achieve a high local concentration of the corresponding osteogenic factor in the area of bone regeneration, polyplexes were included in polylactide particles, which were introduced in chitosan hydrogel or PRP-based fibrin hydrogel. Earlier, in the course of comparative experiments to assess the biocompatibility of hydrogels based on chitosan with different degrees of deacetylation, we determined that chitosan with 39% degrees of deacetylation [18] was optimal. As a result of the polymerization of the fibrinogen contained in PRP, a fibrin clot filled with a large amount of growth factors and cytokines can be obtained, and these play an important role in reducing inflammation and stimulating the migration and differentiation of progenitor cells in the damaged area [21]. In comparison with chitosan hydrogel, the fibrin clot is denser and can reliably hold PLA granules (Figure 1). PRP-based matrices had a positive effect on cell viability and promoted their proliferation in vitro because of the presence of growth factors (Figure 2) and, at the same time, did not cause an immune response in vivo (Figure 3). Similarly, chitosan hydrogels did not cause cytotoxicity in vitro but led to a more noticeable reaction of immune cells during intramuscular implantation (Figure 3), which may be caused by the positive charge of the acyl groups of chitosan [18].

The kinetics of polyplex release from gene-activated matrices is one of the key parameters that determines the efficiency of cell transfection in the defect zone. It is important to ensure a sufficient concentration of vectors a few days after implantation, by the beginning of fibroblast migration and at the formation of granulation tissue, the cells of which will be targets for transfection [23]. PLA granules were chosen for impregnation with plasmid constructs. Polymer scaffolds based on organic acid esters have been previously proven as a suitable material for the deposition of gene constructs, plasmids and viral vectors [15]. In a comparative study of the properties of polylactide materials with different isomeric configurations, it was shown that PLLA have matrix properties that are superior to PDLA [24]. Previously, we obtained highly porous PLA granules with a porosity of 98% [25]. The PLA granules showed high biocompatibility both in vitro and in vivo (Figure 2 and Figure 3). The large surface area of these granules allowed the inclusion of plasmid constructs at high concentrations and ensured their gradual release into the medium over 2 weeks (Figure 4). The introduction of the granules into hydrogel provided a slower release of the vectors from the material, especially from the fibrin clot: 80% of the enclosed DNA was released in 3 weeks (Figure 4). Previously, we selected the optimal ratio of plasmid DNA and TF for efficient transfection of MSCs while maintaining high cell viability [26]. Therefore, based on the release kinetics, the dose of the polyplexes (20 µg/mL pGFP and 40 µL/mL TF) required for impregnation into the matrices was determined. These conditions ensured the efficient transfection of MSCs in vitro (Figure 5). At the same time, the maximum level of *BMP2* gene expression and BMP-2 protein production on day 14 was observed in the presence of matrices based on PLA granules and PRP. The achieved concentration of BMP-2 was sufficient to induce osteogenic differentiation of progenitor cells: in the presence of all the studied matrices, we observed a statistically significant increase in the *Alpl* gene expression and Alpl protein production, as well as noticeable ECM mineralization (Figure 6). These results were higher for TF/pBMP2-PLA/Chit than for TF/pBMP2-PLA/PRP, which may be because of earlier release of polyplexes from the matrices and the triggering of osteogenic cell differentiation.

The achievement of high doses of the osteoinductor BMP-2 using gene-activated matrices was also confirmed in the in vivo experiments. All the studied TF/pBMP2-Matrices demonstrated the formation of young bone tissue 8 weeks after implantation into the rat skull defect area both from the side of the maternal bone and separate foci inside the defect, which was confirmed using histological studies and micro-CT (Figure 7 and Figure 8). The largest amount of newly formed bone was detected during implantation of TF/pBMP2-PLA/PRP, and this was five and seven times greater than that formed with the matrices based on the chitosan and lactide granules, respectively.

The results of the experiment confirm that the use of hydrogels in the composition of matrices promotes bone regeneration, unlike pure polylactides. This is firstly because of the slower release of polyplexes, a process which begins several days later, just after the end of the early inflammatory stage at the site of implantation and migration of mesenchymal cells [23], and secondly, because the fibrous structure of hydrogel matrices contributes to the adhesion of fibroblasts, which begin to form collagen fibers for the future bone matrix. In the PLA group without hydrogels, the number of collagen fibers was significantly lower (Figure 7).

Despite the anti-inflammatory properties claimed by many authors [17], chitosan proved to be more cytotoxic than the PRP-based fibrin clot, which may explain the lower efficiency of neoosteogenesis when the TF/pBMP2-PLA/Chit matrices were implanted into the rat skull defect area compared to that of TF/pBMP2-PLA/PRP. In addition, RPR contains growth factors and anti-inflammatory cytokines [27], and as a result, PRP-based matrices can promote vascular proliferation, cause the migration of osteoprogenitor cells, and reduce the inflammatory response after surgery, thereby accelerating healing. Thus, a comparison of the properties of matrices based on various hydrogels showed that, in terms of gene therapy for bone regeneration, fibrin hydrogel is a component that is preferable to chitosan.

Previously, studies were carried out using several components as matrices for the delivery of plasmids: PLGA microspheres encapsulated with PEI-BMP2 in gelatin sponges [28], PEI-BMP2 embedded in poly(D,L-lactide) coatings on titanium discs [29], a fibrin clot impregnated with BMP2/7 plasmid [30], and chitosan [19]. The effectiveness of many of these components has been shown. Our work demonstrated that the use of PLA granules as a depot for polyplexes in combination with a hydrogel led to new bone formation in critical-size calvarial defects at a period of 8 weeks, which thus corresponds with or exceeds the previously obtained results. Because of the use of vectors with a high level of expression of the target gene and effective transfecting agents for their delivery, it was possible to achieve high levels of BMP-2 protein production both in vitro and in vivo. The combinations of matrices that we developed enabled the kinetics of DNA release to be regulated because of the presence of a viscous hydrogel component, providing a high local concentration of BMP-2 in the area of the bone defect after the active inflammation phase. The fibers of the natural polymers performed an osteoconductive function, supporting the formation of granulation tissue containing fibroblasts. In addition, the active components in PRP increased biocompatibility and enhanced the osteogenic properties of the matrices. The results of our comparative study of matrices based on PLA granules in combination with chitosan or fibrin hydrogel showed that use of matrices based on TF/pBMP2-PLA/PRP was the most effective for bone regeneration.

## 4. Materials and Methods

### 4.1. Matrices

Polylactide granules (PLA) of 0.1–0.4 mm and with a pore diameter of 2–10 μm were freeze-dried using poly-L-lactide with a molecular weight of 200 kDa (4032D, NatureWorks, Plymouth, MN, USA). The porosity of the granules was 98% [25]. PLA granules were sterilized in 70% ethanol for 30 min and then washed in physiological saline (PanEco, Moscow, Russia) for 1 h before testing.

Chitosan (ChitoClear 43040, Primex, Siglufjordur, Iceland) was dissolved in 0.1 M acetic acid for 3 days to obtain a 2.2% solution. A sterile cooled 50% aqueous solution of β-glycerophosphate (Sigma Aldrich, St. Louis, MO, USA) was added in drops to the chitosan solution and constantly stirred at 4 °C to a final concentration of 20%. Chitosan with an initial degree of deacetylation of 65% was re-acetylated in an aqueous-alcoholic medium to obtain chitosan with a degree of deacetylation of 39% [18,26]. Chitosan hydrogel was mixed with PLA granules and incubated at 37 °C for 2 h for gelation.

Blood was collected in tubes with sodium citrate, mixed and centrifuged at 1100 rpm for 10 min at room temperature to obtain platelet-rich plasma (PRP). The supernatant containing platelets and leukocytes was collected and centrifuged at 3600 rpm for 15 min at room temperature. Half of the platelet-poor supernatant was removed, and the precipitate was re-suspended in the remaining volume. Quantitative analysis of blood cells was carried out using a hematological analyzer (Hemalight 1280, Dixion, Russia). The platelet concentration after centrifugation was 4.3 ± 0.6 times higher than it was initially. To obtain a fibrin hydrogel filled with particles, PLA granules were mixed with PRP, a thrombin solution (PZ Cormay, Warsaw, Poland) in 10% calcium chloride solution was added in drops, and the development of the polymerization process was observed within 5 min.

### 4.2. Gene-Activated Matrices

Plasmid DNA with the *BMP2* gene (pBMP2; TaqRFP-N-BMP2, Eurogen, Moscow, Russia) and vectors with the enhanced green fluorescent protein *EGFP* gene (pGFP; pEGFP-C1, Clontech, Mountain View, California, USA) were used. Plasmids were amplified in Escherichia coli cells in an LB broth medium (Sigma Aldrich, St. Louis, MO, USA) with 50 μg/mL kanamycin (Grisp, Porto, Portugal) and isolated using Zymo Research Plasmid Midiprep kit (Zymo Research, Irvine, CA, USA) according to the manufacturer’s protocol. To prepare the polyplexes, 20 µg/mL plasmids and 40 µL/mL transfecting agent TF (Thermo Fisher Scientific, Waltham, MA, USA) were incubated for 20 min at 37 °C. Next, the polyplexes were incubated with sterile PLA granules for 1 h and mixed with chitosan hydrogel or PRP, as described in Section 4.1.

### 4.3. Plasmid DNA Release Kinetics from Matrices

Matrices impregnated with TF/pGFP polyplexes were incubated in saline (PanEco, Moscow, Russia) for 21 d. The DNA concentration was determined via spectrophotometry at a wavelength of 260 nm using a spectrophotometer (NanoDrop OneC, Thermo Fisher Scientific, Waltham, MA, USA).

### 4.4. Cell Cultures

MCSs derived from rat adipose tissue according to a previously developed method [31] were washed with DMEM (PanEco, Moscow, Russia) and 1 mg/mL cefazolin (Biosynthesis, Penza, Russia), and then the tissue was ground and incubated with a 0.1% collagenase type I solution (PanEco, Moscow, Russia) for 1.5 h at 37 °C. Cells were pelleted by centrifugation for 10 min at 1100 rpm at 15 °C and seeded in a DMEM/F12 growth medium (PanEco, Russia) containing 10% fetal bovine serum (FBS; PAA Laboratories, Etobicoke, ON, Canada), 0.584 mg/mL L-glutamine (PanEco, Moscow, Russia), 5000 U/mL penicillin (PanEco, Moscow, Russia) and 5000 µg/mL streptomycin (PanEco, Moscow, Russia) at 37 °C and 5% CO_2_. The growth medium was changed every 3 days.

### 4.5. In Vitro Cytocompatibility Assay

MCSs at 25 × 103 cells/mL were seeded on the bottom of 24-well Transwell system plates (pore diameter 8 µm, SPL Lifesciences, Suwon, Korea) and the matrices were placed in the filters to study their cytocompatibility. Cytotoxicity was assessed using fluorescence microscopy to detect live cells stained with 0.5 μM Calcein-AM (Biotium, Fremont, CA, USA) for 35 min at 37 °C and dead cells stained with 5 μg/mL DAPI (4,6-diamidino-2 -phenylindole) for 10 min at room temperature. Cell viability was assessed using the MTT test after 1 and 7 days. For this, the cells were incubated for 2 h at 37 °C with 0.5 mg/mL 3-(4,5-dimethylthiazol-2-yl)-2,5-diphenyltetrazolium bromide (MTT, PanEco, Moscow, Russia). Formazan crystals were eluted from the cells with DMSO (PanEco, Moscow, Russia), stirred on a shaker for 20 min, and then the optical density was measured on an xMark plate spectrophotometer (Bio-Rad, Hercules, CA, USA) at a wavelength of 570 nm and the background value was subtracted at 620 nm. The results were evaluated in relation to the control, which was taken as 100%.

To assess the cell adhesion of the matrices, MCSs were seeded on the surface of the matrices and after 1 and 3 d, cells were fixed in a 2.5% solution of glutaraldehyde (Panreac, Chicago, IL, USA) for 12 h at 4 °C. The samples were washed in PBS and dehydrated at 4 °C in ethanol battery at increasing concentrations: 50%, 75%, 80%, 90% and in absolute ethanol at the final stage. Then, the samples were air-dried and examined via scanning electron microscopy (SEM) using a Phenom ProX microscope (Phenom, Rotterdam, The Netherlands). The accelerating voltage was 15 kV.

### 4.6. Transfecting Ability of Matrices

TF/pGFP-Matrices were incubated for 7 d with MCSs that were prestained with PKH-26 (red fluorescent cell linker kit, Sigma Aldrich, USA) in accordance with the manufacturer’s instructions, and the transfection efficiency was assessed by detecting the fluorescence of the EGFP protein in cells using a fluorescence Axio Observer D1 microscope with an AxioCam HRc camera (Carl Zeiss Microscopy, GmbH, Oberkochen, Germany).

After 14 d of cultivation with MCSs, the transfecting ability of the TF/pBMP2-Matrices was assessed: for *BMP2* gene expression using RT-PCR and for BMP-2 protein production using ELISA.

RT-PCR was performed in a BioRad iQ cycler thermal cycler (BioRad, USA) with SYBR Green I intercalating dye (Eurogen, Russia). Total RNA was isolated using the RNeasy Plus Mini kit (Qiagen, Hilden, Germany) and cDNA synthesis was carried out using RevertAid (Thermo Scientific, Germany) according to the manufacturer’s protocols. The obtained values of the analyzed genes were normalized to the average values of the reference genes *Gapdh* and *Actβ*. Primers for the studied genes are shown in Table 1.

To assess the production of the BMP-2 protein secreted by MSCs, the medium was collected during the entire period of cell incubation with the studied matrices and stored at −80 °C. Then, all fractions were combined, and the protein was concentrated on Amicon centrifuge filters (Merck KGaA, Darmstadt, Germany) and analyzed via enzyme-linked immunosorbent assay using a Quantikine Elisa kit (R&D Systems, Minneapolis, MN, USA) according to the manufacturer’s protocol. The measurements were carried out on an xMark plate spectrophotometer.

### 4.7. Osteoinduction In Vitro

MSCs were cultivated with TF/pBMP2-Matrices in Transwell system plates for 14 d at 37 °C in a medium supplemented with L-ascorbic acid (Sigma Aldrich, USA) and 2.16 mg/mL β-glycerophosphate (Sigma Aldrich, St. Louis, MO, USA). Half of the medium was replaced every 3 days during the experiment. MSCs in an osteogenic medium were used as a negative control. MSCs in an osteogenic medium supplemented with D3 (F. Hoffmann-La Roche, Basel, Switzerland) were used as a positive control. The efficiency of osteogenic cell differentiation was assessed according to the expression of the *Alpl* gene using RT-PCR, Alpl production using the ICH method and ECM mineralization.

For ICH, cells fixed with 4% formalin solution were incubated with rabbit antiAlpl polyclonal antibodies (Abcam, Cambridge, UK) followed by incubation with antirabbit IgG (H+L) antibodies conjugated with Alexa Fluor 488 (Thermo Fisher Scientific, USA). Cell nuclei were stained with 1 μg/mL DAPI for 10 min. Cells were imaged via fluorescence microscopy using an Axio Observer.D1 microscope and an AxioCam HRc camera.

To detect ECM mineralization, MCSs fixed with cooled 70% ethanol for 30 min at +4 °C were stained with 2% aqueous solution of alizarin red (Sigma Aldrich, St. Louis, MO, USA) pH = 4.1 for 5 min. Next, the culture was washed twice with distilled water to remove unbound dye, and the cells were imaged using light microscopy.

### 4.8. In Vivo Studies

In vivo studies were performed on male Wistar outbred rats weighing 250–300 g. There were six animals in each experimental group. All experiments were approved by the local bioethical committee of Sechenov University (No. PRC-079 from 6 April 2021) in compliance with the Guide for the Care and Use of Laboratory Animals published by the US National Institutes of Health (NIH publication no. 85-23, revised 1996), the European Convention for the Protection of Vertebrate Animals used for Experimental and Other Scientific Purposes, and ISO 10993-2-2009.

Zoletil (Virbac, France) and Xylazine (Interchemie Werken “de Adelaar” BV, Netherlands) were intramuscularly injected for anesthesia at doses of 30 mg/kg and 5 mg/kg, respectively. To prevent postoperative complications, the rats were intramuscularly injected with the antibiotic Ceftriaxone (F. Hoffmann-La Roche, Ltd., Basel, Switzerland) at a dose of 10 μg/kg.

Matrices were implanted 1.5–2 cm deep in the muscle of the posterior thigh to assess biocompatibility. The wound was sutured with Vicryl 5/0 (Ethicon, Guaynabo, PR, USA). Fourteen days after surgery, all rats were euthanized by CO_2_ inhalation, the implantation sites were resected and a histological examination was performed.

The evaluation of osteoinductive properties was carried out using the model of a critical-size calvarial defect. A full-thickness calvarial bone defect was created using a C-reamer trepan of 7.5 mm diameter and 1.5 mm height from the SLA Kit (Neobiotech, Seoul, Korea) with sterile regular saline irrigation. Damage to the superior sagittal sinus was excluded. The matrices were implanted into the formed bone defect. After implantation, the periosteum and skin were sutured with Vicryl 5/0. Fifty-six days after surgery, all rats were euthanized by CO_2_ inhalation, the implantation sites were resected and histological examination and micro-CT were performed.

### 4.9. Histological Assay

Samples were fixed with 10% neutral formalin solution (Labiko, St. Petersburg, Russia) for at least 24 h. Bone biopsy specimens were decalcified in 20% EDTA for 3 weeks. After dehydration in a gradient of alcohols and xylene, the samples were embedded in paraffin. Next, sections were made with a thickness of 5–10 μm. The sections were stained with hematoxylin and eosin (H&E) and Masson’s trichrome staining (Biovitrum, Tomsk, Russia). Histological preparations were analyzed using an Axio Observer D1 microscope with an AxioCam HRc Axioimager M.1 camera. Morphometric analysis was performed on the six serial sections for each sample. The volume of the newly formed bone was evaluated. Morphometric analysis was performed in accordance with the generally accepted recommendations [32,33].

### 4.10. Micro-CT

The new bone formation in the critical-size calvarial defect areas was assessed using a high-resolution micro-CT scanner (SkyScan 1276, Bruker, Kontich, Belgium). Fixed calvarias were placed in a cylindrical specimen holder and scanned at an X-ray voltage of 60 kV. NRecon reconstruction software (Version 1.6.10.2, Bruker, Belgium) was used to generate a 3D reconstruction from a set of scanned images. The obtained images were analyzed using the Dragonfly program (Montreal, QC, Canada).

### 4.11. Statistical Analysis

Statistical analysis and graphing were performed using the SigmaPlot 12.0 software (Systat Soft- ware Inc., Palo Alto, Santa Clara, CA, USA). All data are presented as mean and standard deviation as μ ± SD. Intergroup differences were statistically analyzed using Student’s *t*-test. Differences were considered significant when *p* < 0.05.

## 5. Conclusions

As a result of this work, compositions of gene-activated matrices based on PLA, PLA/Chit and PLA/PRP impregnated with *BMP2* polyplexes were obtained for the first time. The matrices provided cell adhesion, did not cause a cytotoxic effect on MSCs, and PLA/PRP promoted even better cell proliferation. Additionally, no inflammatory reaction was detected during intramuscular implantation of any of the studied matrices.

The use of highly porous PLA granules allowed a high concentration of polyplexes to be included. In addition, PLA granules provided the rapid release of plasmid constructs within 10 days, and matrices based on PLA granules with hydrogels provided a slow gradual release, which was dependent on the density of the gel. All matrices showed transfecting ability and ensured long-term gene expression and the production of target proteins in vitro.

The results showed that the developed gene-activated matrices were safe and effective osteoplastic materials. In addition, PLA granules combined with a hydrogel impregnated with *BMP2* polyplexes had a more pronounced osteoinduction in vivo. Use of PRP-based fibrin hydrogel is preferred because of its additional effect on wound healing. Thus, TF/pBMP2-PLA/PRP can be used in bone regeneration in the future.

## Figures and Tables

**Table 1:** Primers for RT-PCR.

Gene	Nucleotide Sequence
*Actβ*	for GAGATTACTGCCCTGGCTCC rev GCTCAGTAACAGTCCGCCTA
*Alpl*	for TGCCTACTTGTGTGGCGTGA rev CGTGACCTCGTTCCCCTGAG
*BMP* *2*	for ACTACCAGAAACGAGTGGGAA rev GCATCTGTTCTCGGAAAACCT
*Gapdh*	for GCGAGATCCCGCTAACATCA rev CCCTTCCACGATGCCAAAGT

## Data Availability

Not applicable.
